# Besides an ITIM/SHP-1-dependent pathway, CD22 collaborates with Grb2 and plasma membrane calcium-ATPase in an ITIM/SHP-1-independent pathway of attenuation of Ca^2+^_i_ signal in B cells

**DOI:** 10.18632/oncotarget.9794

**Published:** 2016-06-02

**Authors:** Jie Chen, Hong Wang, Wei-Ping Xu, Si-Si Wei, Hui Joyce Li, Yun-Qing Mei, Yi-Gang Li, Yue-Peng Wang

**Affiliations:** ^1^ Department of Cardiology, Affiliated Xinhua Hospital, Shanghai Jiaotong University (SJTU) School of Medicine, Shanghai, China; ^2^ Department of Pediatrics, Affiliated Xinhua Hospital, Shanghai Jiaotong University (SJTU) School of Medicine, Shanghai, China; ^3^ Department of Medicine, University of Massachusetts School of Medicine, Worcester, MA, USA; ^4^ Department of Cardio-Thoracic Surgery, Shanghai Tongji Hospital, Tongji University School of Medicine, Shanghai, China

**Keywords:** B cells activation, calcium signaling, protein-protein interaction, plasma membrane calcium-transporting ATPase 4b

## Abstract

CD22 is a surface immunoglobulin implicated in negative regulation of B cell receptor (BCR) signaling; particularly inhibiting intracellular Ca^2+^ (Ca^2+^_i_)signals. Its cytoplasmic tail contains six tyrosine residues (Y773/Y783/Y817/Y828/Y843/Y863, designated Y1~Y6 respectively), including three (Y2/5/6) lying within immunoreceptor tyrosine-based inhibitory motifs (ITIMs) that serve to recruit the protein tyrosine phosphatase SHP-1 after BCR activation-induced phosphorylation. The mechanism of inhibiting Ca^2+^_i_ by CD22 has been poorly understood. Previous study demonstrated that CD22 associated with plasma membrane calcium-ATPase (PMCA) and enhanced its activity (Chen, J. *et al*. *Nat Immunol* 2004;5:651-7). The association is dependent on BCR activation-induced cytoplasmic tyrosine phosphorylation, because CD22 with either all six tyrosines mutated to phenylalanines or cytoplasmic tail truncated loses its ability to associate with PMCA. However, which individual or a group of tyrosine residues determine the association and how CD22 and PMCA interacts, are still unclear. In this study, by using a series of CD22 tyrosine mutants, we found that ITIM Y2/5/6 accounts for 34.3~37.1% Ca^2+^_i_ inhibition but is irrelevant for CD22/PMCA association. Non-ITIM Y4 and its YEND motif contribute to the remaining 69.4~71.7% Ca^2+^_i_ inhibition and is the binding site for PMCA-associated Grb2. Grb2, independently of BCR cross-linking, is constitutively associated with and directly binds to PMCA in both chicken and human B cells. Knockout of Grb2 by CRISPR/Cas9 completely disrupted the CD22/PMCA association. Thus, our results demonstrate for the first time that in addition to previously-identified ITIM/SHP-1-dependent pathway, CD22 holds a major pathway of negative regulation of Ca^2+^_i_ signal, which is ITIM/SHP-1-independent, but Y4/Grb2/PMCA-dependent.

## INTRODUCTION

CD22 is a surface immunoglobulin-like receptor expressed on mature B cells and a fraction of CD22 is constitutively associated with B-cell receptor (BCR) [1-5]. As an inhibitory co-receptor of the BCR, CD22 plays a critical role in setting signaling thresholds for B-cell activation. Following BCR cross-linking, CD22 inhibits a rapid increment in intracellular Ca^2+^ (Ca^2+^_i_) concentration ([Ca^2+^]_i_) [6-7]. Ca^2+^_i_ signal is required for antigen-induced proliferation and differentiation of B cells into antibody-secreting memory cells. Exaggerated Ca^2+^_i_ signals are seen not only in B cells lacking CD22 but also in SHP-1 (tyrosine phosphatases)-deficient B cells [6-8]. Thus, CD22 and SHP-1 by attenuating Ca^2+^_i_ signals act as negative regulators of B cell autoantibody production and preventers of autoimmunity. CD22 knock-out mice have been shown to be prone to autoimmune diseases, such as lupus [9-11]. On the other hand, up-regulated CD22 function has been reported in patients with immunodeficiency [12, 13].

**Figure 1:**
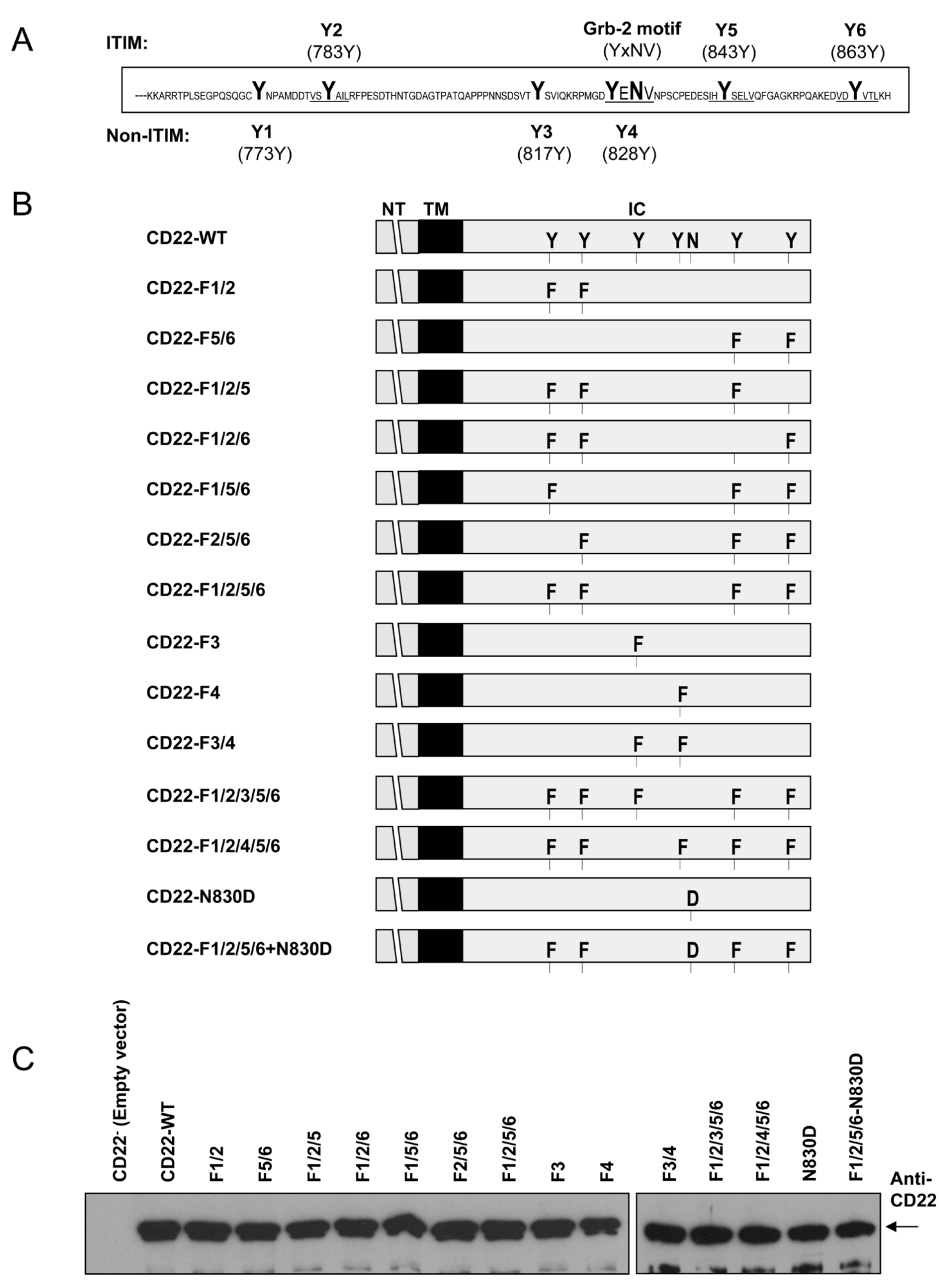
Design and expression of wild type and mutant forms of mouse CD22 **A.** showed CD22′s cytoplasmic tail of 113 amino acids and among them six tyrosines (Y1~Y6) and Grb2 binding motif were marked in capital letters. **B.** showed a list of *CD22-WT* and mutant forms (F, phenyalanine; N, asparagine; D, aspartate) constructs that were cloned into pApuro vector used in this study. **C.** showed the similar expression levels of all these *CD22-WT* and a series of mutant forms in DT40 cells.

CD22′s extracellular immunoglobulin domains binds to α-2,6-linked sialic acid and its cytoplasmic domain consists of 131 amino acid containing six separately-distributed and highly-conserved tyrosine residues at amino acid 773, 783, 817, 828, 843, and 863 (Y773/Y783/Y817/Y828/Y843/Y863, designated Y1~Y6 respectively, see Figure 1A) [1-5, 15, 16]. These tyrosine residues are rapidly phosphorylated by BCR-associated kinase Lyn and Syk upon cross-linking and each phosphorylated tyrosine serves as binding site for distinct effector proteins [17-19]. Y2/5/6 conform to an immunoreceptor tyrosine inhibitory motif (ITIM): I/VxYxxL/V. Competition assay demonstrated that phospho-peptides containing Y2/5/6 could inhibit SHP-1 binding to CD22 [16-18, 29]. Thus, Y2/5/6 is an ITIM and the binding sites for SHP-1 [19, 20]. In addition to SHP-1, ITIMs also recruit signaling molecules such as SHP-2, SHIP (an inositol 5′-phosphatase), Syk (Src family tyrosine kinase), PLC-γ2, PI3K, and SAP [16, 20-24]. SHP-1 is suggested to play a central role in CD22-mediated negative regulation of BCR signaling, including Ca^2+^_i_ signals and mitogen-activated protein kinase (MAPK) cascades through dephosphorylation of various signaling molecules and regulation of PLC-γ2 activity [1, 2, 9, 18, 19, 25-28].

The remaining three tyrosines (Y1/3/4) are classified as non-ITIM. Of them, Y1, as indicated by previous work, does not associate with any effectors [20]. Y3 has been found to recruit Syk [29]. Y4 is mainly the binding site for adaptor protein Grb2 [19, 20, 29-38], in addition to its association to PLC-γ2, PI3K, and adaptor Shc [20, 22, 29]. It is well known that in B cells Grb2 functions as a negative regulator of Ca^2+^_i_ signal. However, the physiological significance of Grb2 direct binding to CD22-Y4 to execute Grb2-mediated Ca^2+^_i_ inhibition remains unclear [32-38].

On the other hand, the knowledge of the pathway that links CD22 to inhibition of the Ca^2+^_i_ signal has been incomplete. Some studies have reported that CD22 may negatively regulate PLC-γ2 activity and thus reduce inositol 1,4,5-triphosphate (IP_3_) production [8, 34]. Previous study reported that CD22 together with SHP-1 enhanced plasma membrane Ca^2+^ ATPase (PMCA)-mediated Ca^2+^_i_ efflux and thus provided a direct mechanism for its negative regulation of [Ca^2+^]_i_ [39]. The association between CD22 and PMCA is dependent on the cytoplasmic tyrosines phosphorylation of CD22 since mutations on all six tyrosines or cytoplasmic tail truncation prevented its binding to PMCA and lost most of the Ca^2+^_i_-dampening function. However, it remains to be determined which tyrosine residue(s) are involved in CD22-PMCA interaction.

In this study, we introduced serial point mutations on the six tyrosine residues in the cytoplasmic tail of CD22 and have analyzed the contributions of each tyrosine residue to Ca^2+^_i_ signal, Ca^2+^_i_ efflux, and CD22/PMCA interaction. Here we present data showing: Mutation of any two of the three ITIM tyrosines (Y2/5/6) has no impact on [Ca^2+^]_i_. Mutation of all three Y2/5/6 contributes to 34.3~37.1% of the [Ca^2+^]_i_ attenuation. Surprisingly, all three ITIM tyrosines do not mediate the interaction with PMCA. Non-ITIM Y4 and Y4-affiliated YEND motif contributes to 69.4~71.7% of the [Ca^2+^]_i_ attenuation and is the PMCA interacting site. Knockout of Grb2 completely disrupts the CD22/PMCA association and most of the CD22-dependent [Ca^2+^]_i_ attenuation. Interestingly, we found that Grb2 is constitutively associated with PMCA in both avian and human B cells, independently of BCR cross-linking. *In vitro* pull-down experiments demonstrate a direct interaction between Grb2 and PMCA. These results indicate CD22 recruits PMCA through Grb2. Thus, our results identify for the first time that in addition to the classic ITIM/SHP-1-dependent pathway, CD22 exerts its negative regulation of Ca^2+^_i_ signal through an ITIM/SHP-1-independent, but Y4/Grb2/PMCA-dependent pathway.

## RESULTS

### Roles of CD22′s ITIM tyrosines in its negative regulation of Ca2^+^_i_ signal, recruitment of SHP-1, and association with PMCA

Previous study showed that either mutation of CD22′s all six tyrosines to phenylalanines or truncation of its cytoplasmic 96 amino acid (Figure 1A) resulted in a complete loss of its negative regulation on Ca^2+^_i_ and its association with PMCA [39]. In this study, we aimed to clarify which individual or a group of tyrosine residues is required for CD22′s negative regulation of Ca^2+^_i_ signal. Figure 1B shows the structure of all CD22 mutants used in this study. We utilized DT40 cells, a CD22-deficient cell line, to make stable transfectants with *Empty vector* (*CD22-*), mouse *CD22-WT*, and a series of mutant forms. As shown in Figure 1C, all mutants showed similar amount of expression of CD22 as compared with *CD22-WT* and thus were suitable for Ca^2+^_i_ and co-IP assay.

**Figure 2:**
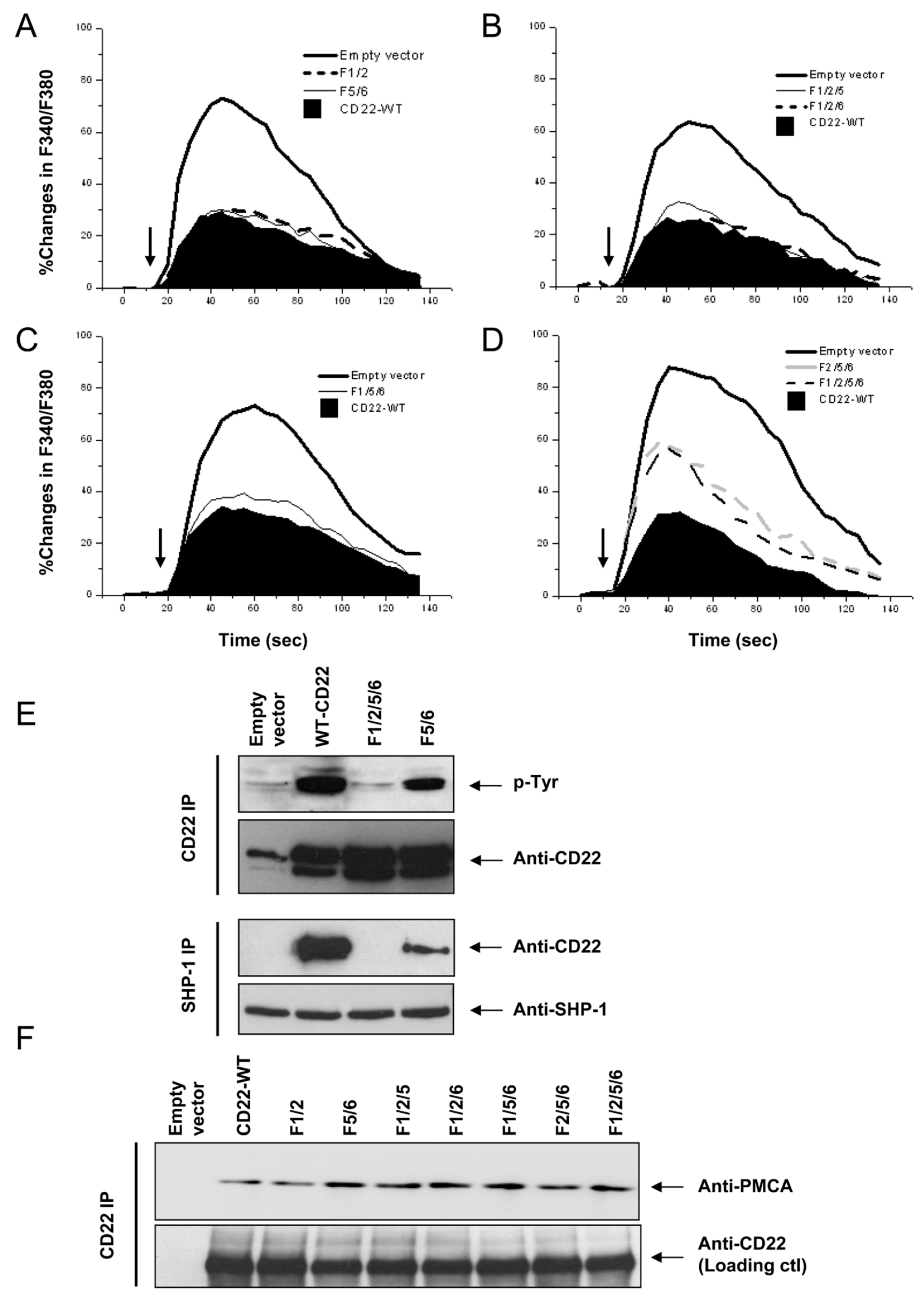
Roles of CD22′s ITIM tyrosines in negative regulation of Ca^**2+**^_**i**_ signal, SHP-1 recruitment, and its association with PMCA DT40 cells were stably transfected with empty vector (*CD22*), *CD22-WT*, and a series of the mutant forms, as indicated. Cells with equal levels of membrane IgM and expressed CD22 were stimulated with 1 μg/ml F(ab’)2 anti-chicken IgM (*arrows*) for 2.5 min. In **A.**-**D.**, the Ca^2+^_i_ responses were measured and compared among cells in the presence of 1 mM EGTA. In *E*, the cells were lysed for IP assay. The panel at top demonstrates IP of CD22 and IB with phorphotyrosine antibody, the second panel showed loading control blot with anti-CD22 antibody, the third panel illustrated IP of SHP-1 and IB with anti-CD22 antibody, and the panel at bottom showed loading control blot with anti-SHP-1 antibody. In *F*, the cells were lysed for IP assay. The *upper* panel showed IP of CD22 and IB with anti-PMCA antibody and the *lower* panel shows loading control blot with anti-CD22 antibody. Results are representative of at least three independent experiments.

The changes in Ca^2+^_i_ were measured in the absence of Ca^2+^_o_ after stimulation with 2 μg/ml F(ab’)_2_ anti-chicken IgM. We first obtained a mean value of the Ca^2+^_i_ parameters, including the Ca^2+^_i_ peak, Δpeak, AUC, ΔAUC, and %ΔAUC, for both *CD22-WT* and *Empty vector* (Table I). Then, experiments were conducted to test the effect of the ITIM tyrosine residues on Ca^2+^_i_ signal. Mutation of CD22 tyrosine residues to phenylalanine as pairs (*F1/2* and *F5/6*) resulted in nominal changes in the Ca^2+^_i_ signals (Figure 2A; Table I). When three tyrosines were mutated (*F1/2/5*, *F1/2/6*, and *F1/5/6*), there was still no significant effect (Figure 2B & 2C; Table I).

**Table I:** Parameters of the lgM-Induced Intracellular Ca^2+^_i_ Signals in DT40 cells

		Ca^2+^_i_ Peak (%)	Δ Peak (%)	AUC (%.s)	Δ AUC (%.s)	%Δ AUC (%)
**Empty (CD22-)**		65.2±9.1##	34.4±0.4##	4825±434##	2738±212##	100##
**CD22-WT**		30.8±5.1	0	2087±104	0	0
**ITIM**	**CD22-F1/2**	31.6±5.0	0.8±0.3	2024±96	−63±13	−2.3±0.4
	**CD22-F5/6**	31.7±4.9	0.9±0.4	1986±96	−101±12	−3.7±0.5
	**CD22-F1/2/5**	31.0±5.6	0.2±0.1	2235±118	148±9	5.4±0.7
	**CD22-F1/2/6**	27.2±3.7	−3.6±0.6	1994±97	−93±7	−3.4±0.4
	**CD22-F1/5/6**	37.6±4.0	6.8±1.2	2202±57	115±14	4.2±0.8
	**CD22-F2/5/6**	46.7±5.9#	15.9±2.1#	3103±194#	1016±47#	37.1±3.1##
	**CD22-F1/2/5/6**	50.2±5.3##	19.4±2.3##	3026±170#	939±47#	34.3± 3.2##
**Non-ITIM**	**CD22-F3**	41.4±6.2#	10.6±1.1	2320±149	233±43	8.5±2.3
	**CD22-F4**	58.8±5.6##	28.0±2.5##	3987±240#	1900±121##	69.4±4.9##
	**CD22-F3/4**	60.3±7.4##	29.5±3.1##	4080±245##	1993±89##	70.8±4.7##
**ITIM and Non-ITIM**	**CD22-F1/2/3/5/6**	51.4±11.0##	20.6±1.9##	3196±116#	1109±57#	40.5± 5.6##
	**CD22-F1/2/4/5/6**	68.6±8.6##	37.8±2.9##	4896±194##	2809±210##	101.6±7.6##
	**C022-N830D**	64.1±5.1##	33.3±3.1##	4160±111##	2073±188##	71.7± 5.1##
	**CD22-F1/2/5/6+N8300**	67.1±7.7##	36.3±3.4##	4773±385##	2686±276##	98.1±6.3##
**WT DT40**		49.3±7.4	17.9±5.3^@^	3647±131^@^	1229±63^@^	-
**Grb2^−/−^**	**Grb2^−/−^**	65.6±4.9^*^	30.3±5.1^*^	4894±227^*^	2611±179^*^	-
	**Grb2^−/−^/CD22^+^**	33.2±6.1	0	2324±96	0	-
**Grb2^−/−^/Grb2^+^**	**Grb2^−/−^/Grb2^+^**	61.1±5.5^**^	40.7±5.4^**^	4983±185	2801±176^**^	-
	**Grb2^−/−^/Grb2^+^/CD22^+^**	20.4±4.7	0	1783±81	0	-

Ca^2+^_i_ Peak (%) = the peak level of Ca^2+^_i_;AUC = the area under the Ca2^+^_i_ curve

Δ Peak(%)= CD22-mutant's Peak − CD22-WT's Peak; = WT DT40′s Peak − Grb2^−/−^/CD22^+^'s Peak; = Grb2^−/−^'s Peak − Grb2^−/−^/CD22^+^'s Peak; = Grb2^−/−^/Grb2^+^'s Peak − Grb2^−/−^/Grb2^+^/CD22^+^'s Peak

Δ AUC (%.s) = CD22-mutant's AUC − CD22-WT's AUC; = Grb2^−/−^'s AUC − WT DT30′s AUC; = Grb2^−/−^'s AUC − Grb2^−/−^/CD22^+^'s AUC; = Grb2^−/−^/Grb2^+^'s AUC − Grb2^−/−^/Grb2^+^/CD22^+^'s AUC

%Δ AUC (%) = (CD22-mutant's AUC − CD22-WT's AUC) / (Empty vector's AUC − CD22-WT's AUC) × 100%

# *p*<0.05

## p<0.01 *vs* CD22-WT

@ *p*<0.05 *vs* Grb2^−/−^

* *p*<0.01 *vs* Grb2^−/−^/CD22^+^

** *p*<0.01 *vs* Grb2^−/−^/Grb2^+^/CD22^+^

Mutation of all three classic ITIMs Y2/5/6 (*F/2/5/6*) or in combination with Y1 mutation (*F1/2/5/6*) significantly potentiated Ca^2+^_i_ signal (Figure 2D; Table I). There was no significant difference between *F2/5/6* and *F1/2/5/6* (*p* > 0.05). These results indicated that ITIM Y2/5/6 account for 34.3~37.1% of the CD22′s negative regulation on Ca^2+^_i_ signal and that Y1 is dispensable.

ITIM Y2/5/6 has been shown to be binding sites for SHP-1, which has been recognized as the most important down stream effector of CD22 [16-19]. Therefore, we next investigated CD22 phosphorylation using 10 μg/ml F(ab’)_2_ anti-chicken IgM to activate BCR and compared the CD22-SHP-1 interaction among the ITIM mutants. As shown in Figure 2E, *F1/2/5/6* has almost totally abolished BCR-induced tyrosine phosphorylation and disrupted the CD22/SHP-1 interaction, as compared to *CD22-WT*. *F5/6* also caused a mild decrease in tyrosine phosphorylation signal and a moderate decrease in CD22/SHP-1 interaction. These results confirmed that ITIM residues Y2/5/6 account for almost complete binding to SHP-1, as suggested by previous studies [18, 19].

Next we examined the effect of ITIM tyrosine residues on the association between CD22 and PMCA by co-IP assay (Figure 2F). Surprisingly, CD22 mutants, including *F1/2*, *F5/6*, *F1/2/5*, *F1/2/6*, *F1/5/6*, *F2/5/6*, and *F1/2/5/6*, all demonstrated a completely intact association with PMCA, as compared with *CD22-WT*. These results suggested that all three ITIM Y2/5/6 do not mediate the binding with PMCA.

### Role of CD22′s non-ITIM Y3, Y4, and Y4-affiliated Grb2 binding motif in its negative regulation of Ca2^+^_i_ signal, Ca2^+^_i_ extrusion, recruitment of SHP-1, and association with PMCA

**Figure 3:**
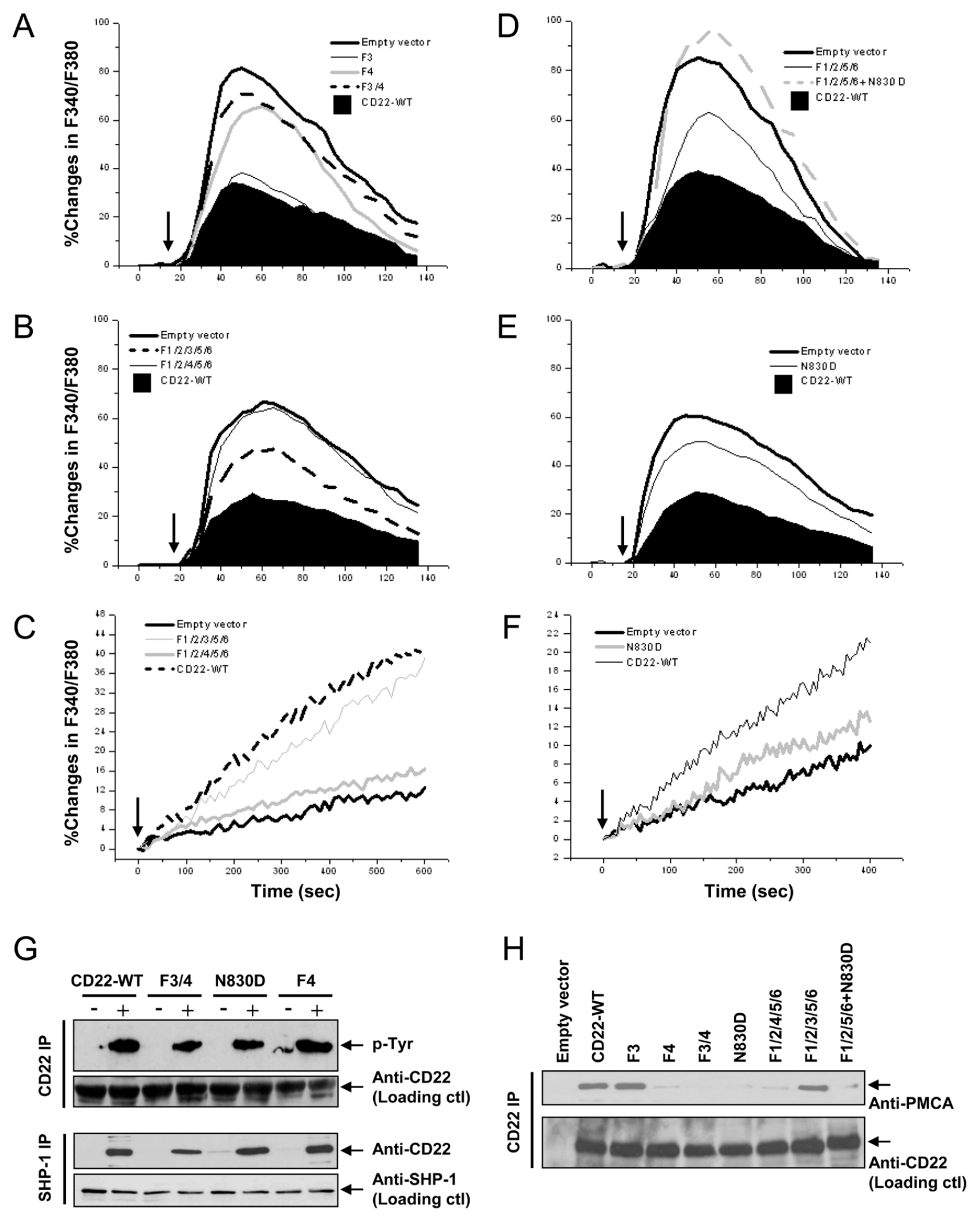
Roles of CD22′s non-ITIM residues Y3, Y4, and Y4-affiliated Grb2 binding motif in negative regulation of Ca^**2+**^**_i_** signal, Ca^2+^_**i**_ efflux, SHP-1 recruitment, and its association with PMCA DT40 cells were stably transfected with empty vector (*CD22*), CD22-WT, and a series of mutant forms, as indicated. Cells with equal levels of membrane IgM and expressed CD22 were stimulated with 1 μg/ml F(ab’)2 anti-chicken IgM (*arrows*) for 2.5 min. In **A.**, **B.**, **D.**, and **E.**, the Ca^2+^_i_ responses were measured and compared among cells in the presence of 1 mM EGTA. **C.** and **F.** compared the Ca^2+^_i_ efflux. In **G.**, the cells were lysed for IP assay. The panel at top demonstrates IP of CD22 and IB with phorphotyrosine antibody, the second panel shows loading control blot with anti-CD22 antibody, the third panel IP of SHP-1 and IB with anti-CD22 antibody, and the panel at bottom shows loading control blot with anti-SHP-1 antibody. In **H.**, the cells were lysed for IP assay. The upper panel demonstrates IP of CD22 and IB with anti-PMCA antibody and the lower panel shows loading control blot with anti-CD22 antibody. Results are representative of at least three independent experiments.

Is the remaining > 60% negative regulatory effect on [Ca^2+^]_i_ caused by the non-ITIM Y3 and Y4? The following experiments were performed to answer this question. As shown in Figure 3A and Table I, *F3* has a little but insignificant effect. However, *F4* demonstrated a significant potentiation in Ca^2+^_i_ signal. *F3/4* also illustrated a remarkable potentiation on Ca^2+^_i_ signal.

To further identify whether Y3 and Y4 mediate this ITIM-independent residual inhibition through working together with ITIM Y2/5/6, we mutated an additional Y3 or Y4 on the *F1/2/5/6* background (*F1/2/3/5/6* or *F1/2/4/5/6*). As expected, *F1/2/3/5/6* had no significant additional effect, as compared with *F1/2/5/6* (Figure 3B). However, *F1/2/4/5/6* remarkably potentiated Ca^2+^_i_ signal. These results ruled out the possibility that Y4 potentiated Ca^2+^_i_ signal by collaboration with ITIM. It is more likely that Y4 works ITIM-independently.

Meanwhile, we also measured Ca^2+^_i_ extrusion, which is a more direct index reflecting PMCA's activity, using same method as in the previous study [39]. In accordance to the changes in Ca^2+^_i_ in Figure 3B, *F1/2/3/5/6* had only mild effect on Ca^2+^ extrusion (19.4±3.2% at 300sec), as compared with *CD22-WT* (21.4±2.7% at 300sec; *p* > 0.05). *F1/2/4/5/6* significant inhibited Ca^2+^_i_ efflux (7.8±1.1% at 300sec; *p* < 0.05 *vs CD22-WT*; Figure 3C), getting close to the *Empty vector*'s level (6.5±1.3% at 300sec; *p* > 0.05 *vs CD22-WT*). These results further indicated that between Y3 and Y4, Y3 is dispensable, but Y4 is a key residue responsible for CD22′s ITIM-independent negative regulatory function. Furthermore, our results demonstrated that although both ITIMs and non-ITIM were responsible for the negative regulation of Ca^2+^ signal, non-ITIM Y4 is more important than ITIM Y2/5/6 and contributed to 69.4~71.7% of the CD22′s negative regulation.

Previous studies have shown that Y4 recruits Grb2 when Y4 is phosphorylated [19, 20]. The SH2 domain of Grb2 directly binds to phosphorylated Y4 with an asparagine (N) in the +2 position (N830) to form a Grb2 binding motif of pYXNV. Therefore, the binding could be disrupted not only by mutation of Y4 to *F4*, but also by mutation of the N830 to aspartate (D; *N830D*) [40]. To further verify whether Y4 and Y4-associated Grb2 binding motif play a significant role in the negative regulation of Ca^2+^_i_ signal, we made two mutants of CD22: a single N830→D (*N830D*) and *N830D* on the *F1/2/5/6* background (*F1/2/5/6+N830D*). *N830D* significantly diminished *CD22-WT*'s inhibitory function on Ca^2+^_i_ signal (Figure 3E; Table I) and Ca^2+^_i_ extrusion (9.68±0.9% at 300sec; *p* > 0.05 *vs CD22-WT*; Figure 3F). Furthermore, *F1/2/5/6+N830D* almost totally abolished CD22′s inhibitory function (Figure 3D; Table I), similar to *F1/2/4/5/6*'s effect (Figure 3B). These results further revealed that Y4 and Y4-associated Grb2 binding motif are necessary for CD22′s inhibitory function.

Next we examined 10 μg/ml IgM-activated CD22 phosphorylation and compared the CD22-SHP-1 interaction among the various non-ITIM mutants. As shown in Figure 3G, *F3/4*, *F4*, and *N830D* had no significant effect on IgM-activated CD22 phosphorylation, as compared to *CD22-WT*. *N830D* and *F4* held a completely intact, while *F3/4* had a mild decreased association with SHP-1, as compared with *CD22-WT*. These results indicated that both *F4* and *N830D* had no significant effects on both tyrosine phosphorylation and SHP-1 recruitment.

We then determined the impacts of Y3 and Y4 and Y4-affiliated Grb2 binding motif on the association between CD22 and PMCA by co-IP assay (Figure 3H). Compared to *CD22-WT*, only *F3* and *F1/2/3/5/6* demonstrated a totally intact association with PMCA. All other Y4-related mutants, including *F4*, *F3/4*, *F1/2/4/5/6*, *N830D*, and *F1/2/5/6+N830D* demonstrated an almost complete loss of association with PMCA.

### Y4, Y4-affiliated Grb2 binding motif, and Grb2 are essential for CD22/PMCA pathway

It has been shown that phosphor-Y4 (pY4) is the binding site for Grb2 after BCR activation [19, 20, 29-38]. To investigate the role of Grb2 on the CD22/PMCA pathway, we made a Grb2^−/−^ DT40 cell line by CRISPR/Cas9 method. We obtained two successful clones, one with single base pair deletion and another with 5 base pair deletions in the target region of the Grb2 gene. In both cases, the deletions resulted in a frame shift and thus truncation of Grb2 protein (Supplementary Figure 1S).

**Figure 4:**
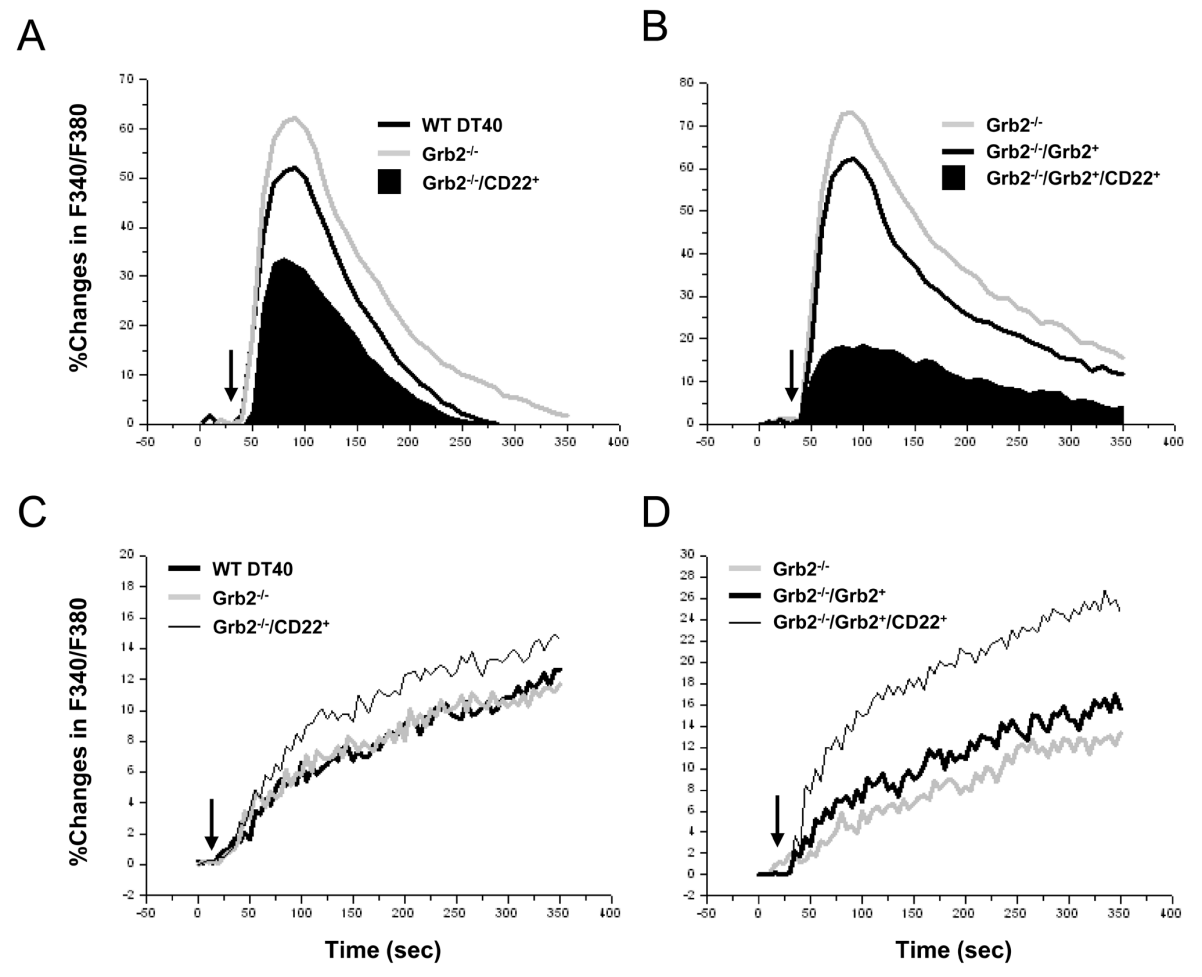
Roles of Grb2 in CD22′s negative regulation of Ca^2+^_i_ Signal and Ca^2+^_i_ efflux *Empty vector* (*CD22*) or *CD22-WT* (*CD22*) was stably transfected into Grb2^−/−^ DT40 cells **A.**-**D.** Grb2^−/−^ DT40 cells were further rescued by transfection of chicken Grb2 (*B*, *D*), with or without co-transfection of CD22. The Ca^2+^_i_ (*A*, *B*) and Ca^2+^_i_ efflux (*C*, *D*) were measured in IgM-matched cells after stimulation with 2 μg/ml anti-chicken IgM (*arrows*) in the presence of 1 mM EGTA (*A*, *B*) and in the efflux buffer (*A*, *B*). Results were representative of at least three independent experiments.

After comparison of the IgM expression, the clone with 5 base pair deletions was selected for studying the impacts of Grb2 null(*Grb2^−/−^*) on the CD22′s negative regulation of Ca^2+^_i_ signal. Compared to *WT DT40* cells, Grb2^−/−^ cells demonstrated a potentiated Ca^2+^_i_ signal (Figure 4A; Table I) and an indistinguishable difference in Ca^2+^_i_ extrusion. However, compared to *Grb2^−/−^* cells, expression of *CD22-WT* in Grb2^−/−^ cells (*Grb2^−/−^/CD22^+^*) significantly inhibited Ca^2+^_i_ signal, with a mildly increased Ca^2+^_i_ extrusion (10.3±2.3% *vs* 13.1±2.5% at 300sec, *p* > 0.05; Figure 4C).

As a rescue experiment, we transfected full-length WT chicken Grb2 cDNA back to the Grb2^−/−^ cells, with (*Grb2^−/−^/Grb2^+^/CD22^+^*) or without (*Grb2^−/−^/Grb2^+^*) co-expression of *CD22-WT*. Compared to *Grb2^−/−^* cells, *Grb2^−/−^/Grb2^+^* cells showed an inhibited Ca^2+^_i_ signal (Figure 4B; Table I) and mildly increased Ca^2+^_i_ efflux (13.2±2.8% *vs* 15.8±2.7% at 300 sec, *p* > 0.05; Figure 4D). However, compared to *Grb2^−/−^/Grb2^+^* cells, *Grb2^−/−^/Grb2^+^/CD22^+^* cells significantly regained its inhibitory function on Ca^2+^_i_ signal (Figure 4B; Table I) and stimulatory function on Ca^2+^_i_ efflux (24.5±3.1% at 300sec; *p* < 0.05; Figure 4D).

**Figure 5:**
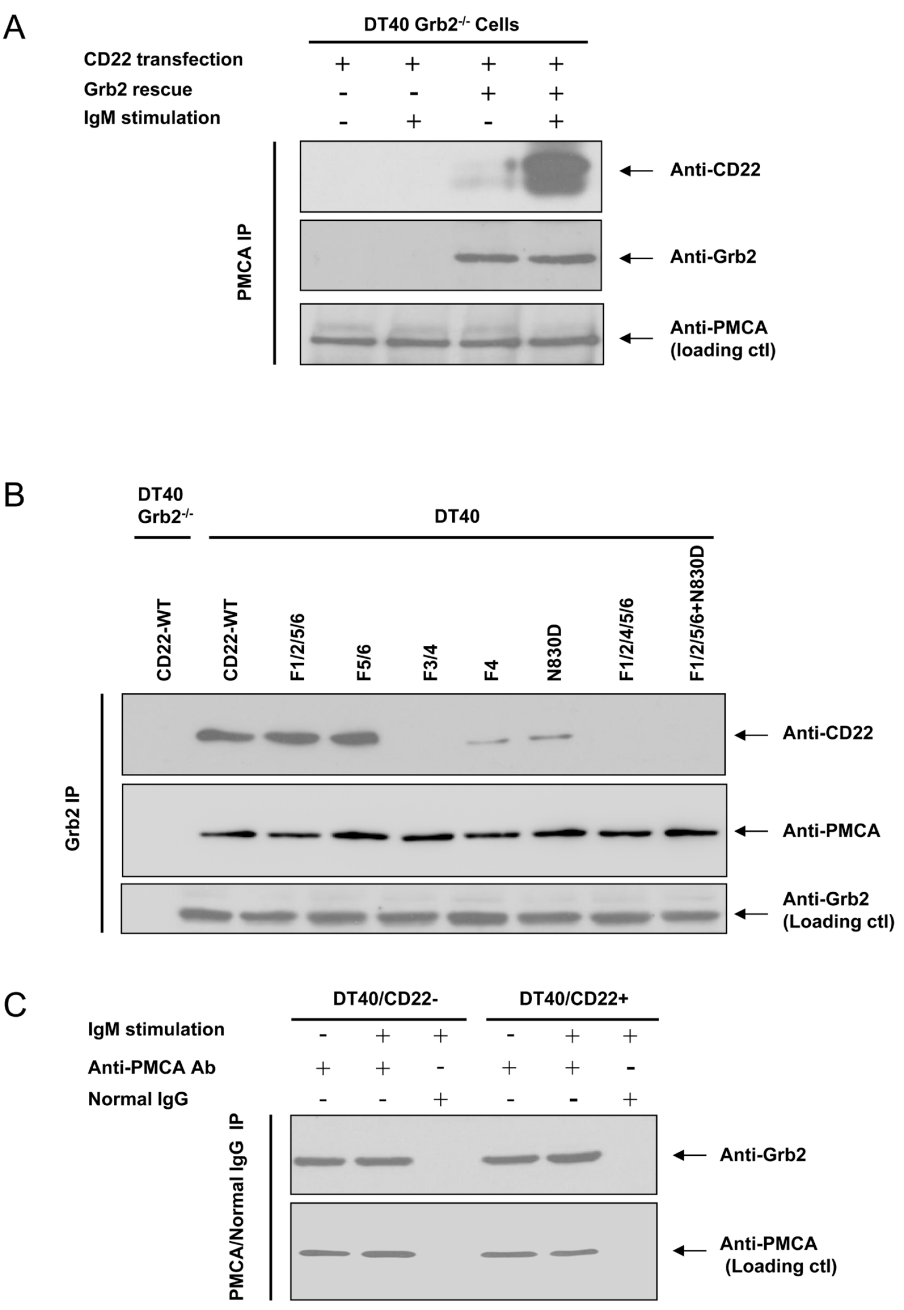
Grb2 is necessary for PMCA and CD22 association In **A.**, *CD22-WT* was stably transfected into Grb2^−/−^ DT40 cells (the 1^st^ ~ 4^th^ lane), with (the 3^rd^ and 4^th^ lane) or without (the 1^st^ and 2^nd^ lane) co-transfection of chicken Grb2. After with (+) or without (−) stimulation of 1 μg/ml F(ab’)2 anti-chicken IgM for 2.5 min, the cells were harvested for IP study. The upper panel demonstrates IP of PMCA and IB of anti-CD22 antibody, the middle panel shows IB of Grb2, and the lower panel shows loading control blot with anti-PMCA antibody. In **A.**, the Grb2^−/−^ (the 1^st^ lane) or normal (the remaining lanes) DT40 cells were transfected with *CD22-WT* or mutant forms, as indicated. All cells were stimulated with 5 μg/ml F(ab’)2 IgM for 2.5 min and harvested for IP study. The upper panel demonstrates IP of Grb2 and IB of CD22. The middle panel shows IB of PMCA. The lower panels show the loading control blot with anti-Grb2 antibody. In **C.**, DT40 cells were stably transfected with Empty vector (*CD22-*), *CD22-WT*, with (+) or without (−) stimulation of 1 μg/ml F(ab’)2 IgM for 2.5 min. Cells lysate were obtained for IP experiment. The upper panel showed IP with PMCA antibody or normal mouse IgG and IB of Grb2. The lower panel showed loading control blot with anti-PMCA antibody.

Next, we asked if CD22/PMCA association is dependent on Grb2? We transfected mouse *CD22-WT* into Grb2^−/−^ DT40 cells and found that CD22 and PMCA failed to associate with each other, either with or without BCR activation (Figure 5A). We further transfected chicken Grb2 cDNA back to Grb2^−/−^ DT40 cells, CD22 regained its association with PMCA (Figure 5A). These results indicated that the association between CD22 and PMCA required Grb2 as a mediator.

In order to further test whether Grb2 provided a bridge for CD22 and PMCA association and compare the effect of ITIM and non-ITIM tyrosines on this BCR-activation-induced formation of the CD22/Grb2/PMCA complex, we performed co-IP assays using anti-Grb2 antibodies from lysate of Grb2^−/−^ or normal DT40 cells which were transfected with *CD22-WT* or serial CD22 mutants. In Grb2^−/−^ cell lysate we failed to precipitate Grb2 and co-precipitate both CD22 and PMCA. In normal DT40 cell lysate, results showed that CD22/Grb2/PMCA complex were intact from lysates of *CD22-WT* and its *F1/2/5/6* and *F5/6* mutants, but were disrupted in the mutants of *F3/4*, *F4*, *N830D*, *F1/2/4/5/6*, and *F1/2/5/6+N830D* (Figure 5B). These results further demonstrated that the CD22/Grb2/PMCA complex is formed through BCR activation-induced Y4 phosphorylation and Y4-assocaited Grb2-binding motif to recruit Grb2, which mediates the association of CD22 and PMCA. These results also further excluded the possibility that ITIM and SHP-1 are involved in mediating the interaction.

Is the association between PMCA and Grb2 also BCR activation-dependent? Co-IP approach from normal DT40 cell lysate showed that PMCA consistently associates with Grb2, regardless of the presence or absence of CD22, and regardless of with or without BCR activation (Figure 5C).

**Figure 6:**
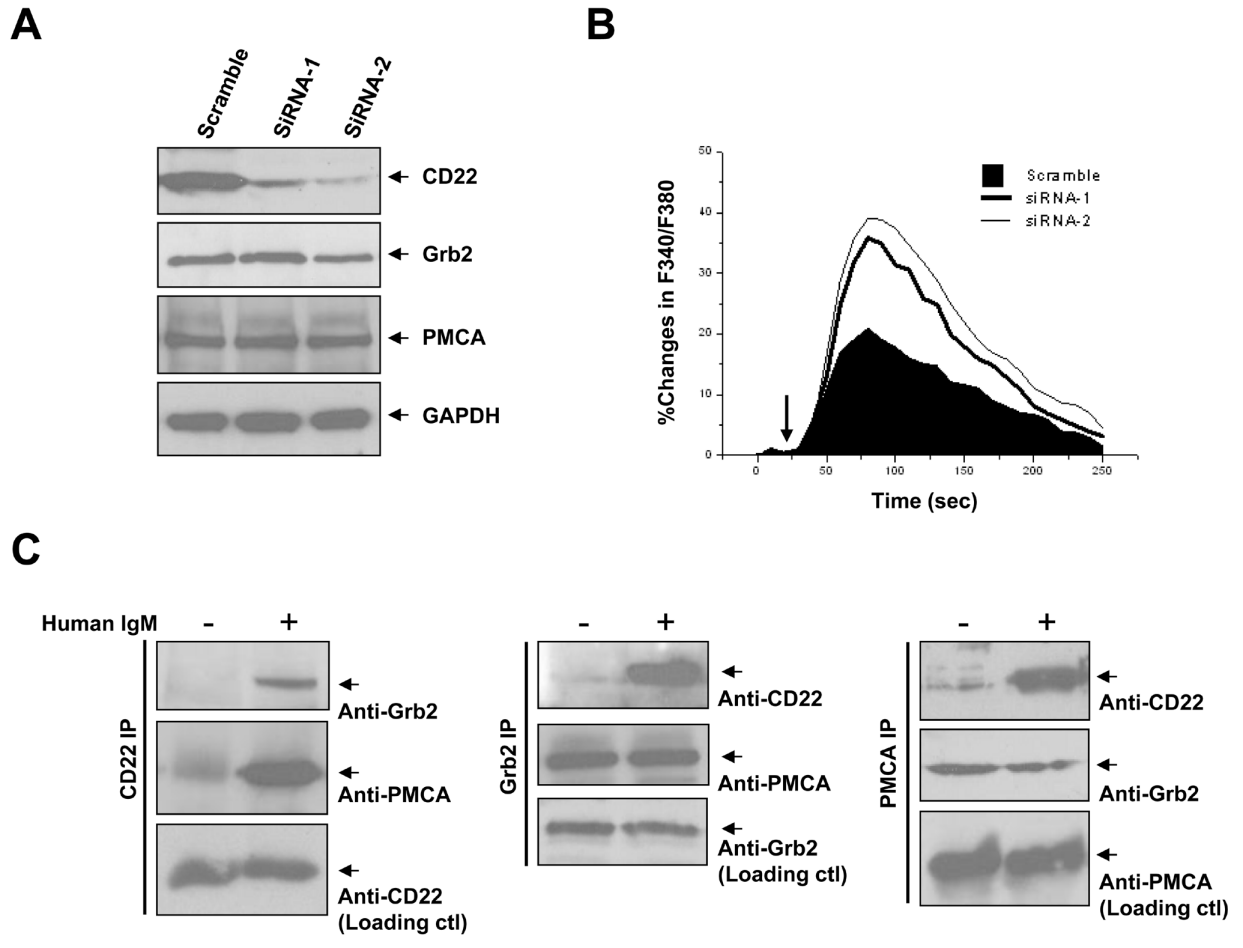
Negative regulation of Ca^**2+**^_**i**_ signaling by CD22 and BCR activation-dependent CD22/Grb2/PMCA module formation in human B cells In **A.** and **B.**, Daudi cells were transfected with scramble oligonucleotides and specific siRNA-1 and −2 towards CD22. Between 72~96 hours after transfection, cells were used for experiments. In *A*, cells were lysed for checking expression of CD22 (*top*), Grb2 (*middle*), and PMCA (*bottom panel*). In **B.**, Ca^2+^_i_ responses were measured and compared in the presence of 1 mM EGTA after treatment of 5 μg/ml F(ab’)2 anti-human IgM (*arrows*). In **C.**, freshly-isolated normal human B cells (see *Material and Methods*) were lysed for IP assay, with (*+*) or without (*-*) pretreatment of 5 μg/ml F(ab’)2 anti-human IgM (+). Data showed IP of CD22 (*left*), Grb2 (*middle*), and PMCA (*right*), and IB three of them as indicated. Results were representative of at least three independent experiments.

Above work was performed in the chicken DT40 cells, which is easily accessible to molecular manipulations. However, the physiological relevance may be limited because of its immaturity. We next to ask whether the pathway of CD22/Grb2/PMCA to inhibit Ca^2+^_i_ signal is also working in human mature B cells? We first confirmed the expression of the three proteins in Daudi cells, a human Burkitt's lymphoma cell-line (data not shown). We then transfected specific siRNA against CD22 into Daudi cells and successfully inhibited most of the CD22 expression (Figure 6A). We compared the Ca^2+^_i_ signal with or without CD22 inhibition. As a result, inhibition of CD22 significantly potentiated the Ca^2+^_i_ signal (Δpeak 18.2±3.5%; ΔAUC 3090±148%.s; both *p* < 0.01), as compared to the cells transfected with scramble oligonucleotides (Figure 6B).

To determine whether CD22/Grb2/PMCA can form complexes in normal human B cells, we next isolated peripheral B cells, cultured for a short time, stimulated with 5 μg/ml anti-human F(ab’)2 IgM, made cell lysate, and performed immuno-precipitation experiments. We found that the complex of CD22/Grb2/PMCA was associated together only after BCR activation, independent of the sequence of proteins precipitated (Figure 6C). However, Grb2 and PMCA, but not CD22, were always bound to each other even without BCR cross-linking. These results were consistent with above observations in DT40 cells and indicated that CD22/Grb2/PMCA complexes can be observed in normal human B cells.

**Figure 7:**
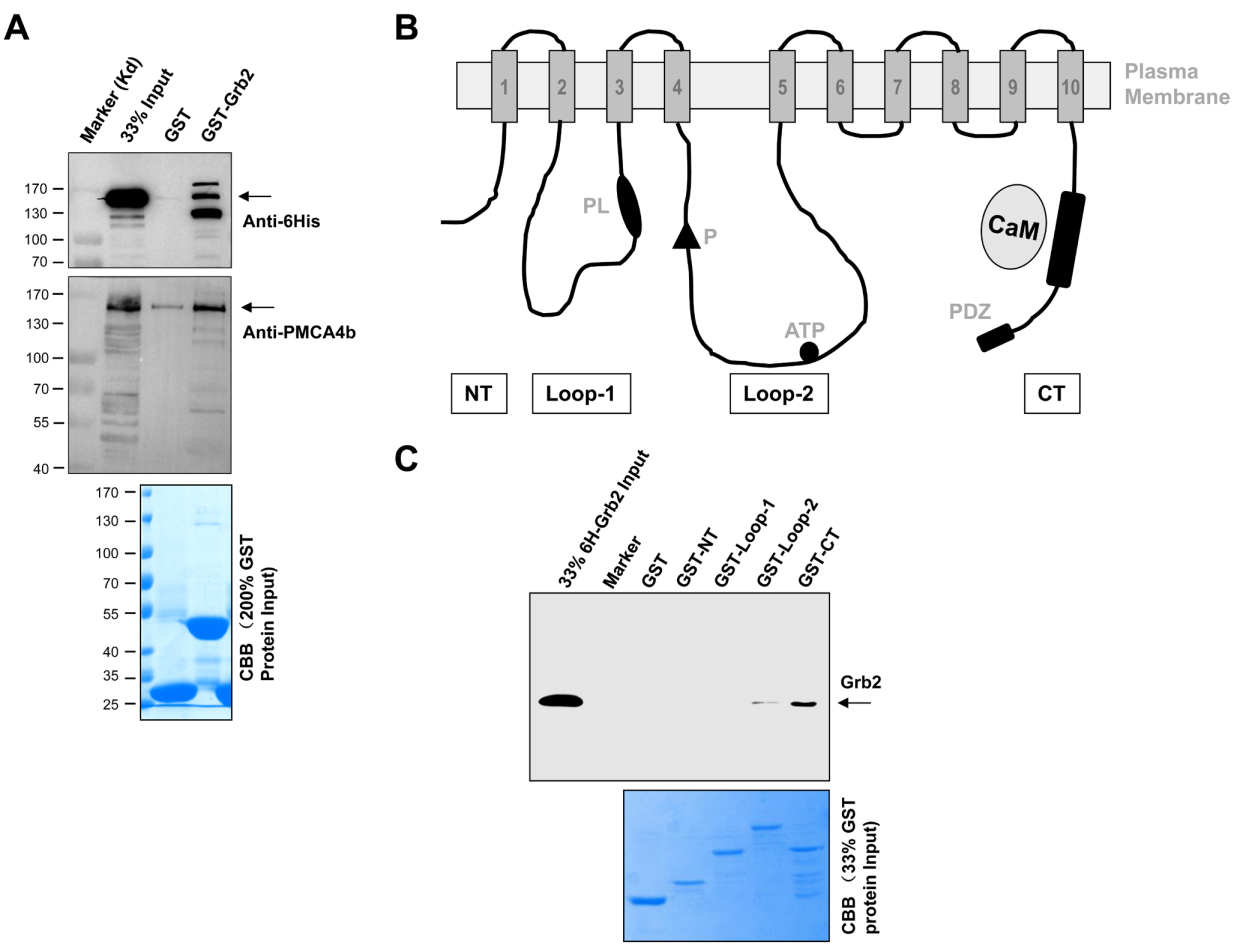
Direct interaction between Grb2 and PMCA **A.** showed the results of GST-Grb2 pulling-down full-length PMCA4b that was over-expressed in 293T cells, shown by *Western* blotting of anti-6His (*top*) and anti-PMCA4b (*middle*) antibodies and by CBB staining of GST and GST-Grb2 inputs (*bottom*). **B.** is the domain structure of the PMCA4b. The membrane-spanning regions are numbered and shown as shaded boxes. Labels are the amino- (NT) and carboxyl-terminal (CT) ends, the obligatory aspartyl-phosphate (P), ATP binding region, the phospholipid-sensitive region (PL), the calmodulin binding region (CaM), and the PDZ-binding motif. **C.** showed the results of GST-fusion proteins pulling-down 6His-Grb2, revealed by *Western* blotting of anti-Grb2 (*upper*) antibodies and by CBB staining (*lower panel*) of the protein inputs. Results were representative of at least three independent experiments.

At last, how does PMCA associate with Grb2, directly or indirectly? In order to answer this key question, we expressed human Grb2 in bacteria and full-length human PMCA4b in HEK293T cells. As shown in Figure 7A, purified GST-Grb2 specifically pulled down PMCA4b. The molecule of the human PMCA4b has four major cytoplasmic fragments and they are NT (AA 1-92), Loop-1 (AA 172-356), Loop-2 (AA 428-840), and CT (AA 1048-1241; Figure 7B). We further expressed and purified these four GST-fusion fragmental proteins and full-length 6His-Grb2 in bacteria. In pull-down assay, we found that, only its CT bound with full-length 6His-Grb2 (Figure 7C). These results demonstrated that Grb2 interacted directly with PMCA4b.

## DISCUSSION

The previous study demonstrated that six tyrosines present at CD22 cytoplasmic domain and their phosphorylation are prerequisites for its interaction with PMCA [39]. In this study, by screening the changes in Ca^2+^_i_ signaling, Ca^2+^_i_ efflux, and co-IP assay among a series of CD22 mutants, we found that pY4 is the key for its association with PMCA. We also found that the interaction between CD22 and PMCA is indirectly mediated by Grb2. Grb2 is constitutively associated with PMCA in both DT40 and human B cells. *In vitro* pull-down experiments using purified proteins demonstrate a direct interaction between Grb2 and PMCA. Furthermore, we identified the CD22/Grb2/PMCA pathway as a major route for its negative regulation of Ca^2+^_i_ signal since this pathway contributes to 69.4~71.7% of the Ca^2+^_i_ inhibition. CD22′s ITIM/SHP-1-dependtent pathway accounts for only 34.3~37.1% of the Ca^2+^_i_ inhibition.

Grb2 is an adaptor protein that interacts with numerous receptors and intracellular signaling molecules. It is widely expressed and is essential for multiple cellular functions. Subcellular distribution of Grb2 in B cells could be both in cytoplasm and on plasma membrane [34]. Grb2 composes of a single SH2 domain, flanked by two SH3 domains with no catalytic domains. Its SH2 domain binds to pY-containing peptides on receptors or scaffold proteins with a preference for pYXNV. Stork *et al*. have shown that Grb2 is a negative regulator of Ca^2+^_i_ signaling in DT40 cells [32, 34]. Since 1999, several studies have demonstrated that following BCR cross-linking CD22 recruits Grb2 through its pY4 and Grb2′s SH2 domain binds directly to CD22′s pYENV motif [19, 20, 29].

However, the physiological significance of Grb2 recruitment to CD22′s pY4 has been unclear [32-38]. In this study we identified for the first time that the significance is to inhibit Ca^2+^_i_ signal by recruiting PMCA. Grb2 directly binds to PMCA C-terminus, which contains neither SH2 domain nor other recognized tyrosine-binding motif [41], but has a calmodulin binding site and a Ca^2+^-dependent PDZ domain to respectively mediate Ca^2+^-calmodulin binding and other protein-protein interactions (Figure 7B) [42].

The domain(s) mediating the binding of Grb2 to PMCA C-terminus is not addressed in this study. Grb2′s

N-terminal SH3 domain preferentially binds to proline-rich peptides-containing proteins, such as SOS (a Ras-guanine nucleotide exchange factor) [43]. We thus searched and did not find any remarkable proline-rich regions in the whole sequence of PMCA4b. Grb2′s C-terminal SH3 domain binds to peptides conforming to a P-X-I/L/V/-D/N-R-X-X-K-P motif that allows it to specifically bind to proteins such as Gab-1 [8]. In B cells, Grb2′s C-terminal SH3 domain has been identified to be critically important for its inhibitory regulation of Ca^2+^ signal [32]. Therefore, Grb2 may directly bind to PMCA through its C-terminal SH3 domain. Further study is needed to address this issue.

Next, whether CD22/Grb2/PMCA module associates with effector proteins is still unknown. In B cells, Grb2 at least binds to SOS and Dok-3 (hematopoietic protein downstream of kinase-3), both of which have been reported to be involved in the negative regulation of BCR signaling and contain a typical YXNV domain [32, 34, 43].

SOS may associate with the CD22/Grb2/PMCA module. It is best known that Grb2 constitutively binds with its N-terminal SH3 domains to the proline-rich regions of SOS to form a stable complex to activate Ras [43]. SOS-mediated exchange of GDP for GTP activates Ras, which in turn activates the protein kinase Raf and subsequently the extracelluar signal-regulated kinase (Erk), which is one of the MAPK pathways. Grb2 may be recruited to the membrane by SOS through SH2 domain of Grb2. In this case, CD22 or SOS may compete for the binding to Grb2, since Grb2 has only one SH2 domain. Therefore, the most possible context that depicts SOS's association with the CD22/Grb2/PMCA module is that Grb2′s N-terminal SH3 domain binds to proline-rich domain-containing SOS [43], Grb2′s C-terminus SH3 domain binds to PMCA, and Grb2′s SH2 domain bind to CD22.

Dok-3 may facilitate the CD22/Grb2/PMCA module formation by recruiting Grb2 to plasma membrane. Dok-3 is an adaptor protein highly expressed in B cells and plays a significant role in the negative regulation of BCR signaling [34]. Dok-3 is permanently tethered at the inner leaflet of the plasma membrane *via* its pleckstrin homology (PH) domain and Grb2 binds *via* its SH2 domain to tyrosine phosphorylated Dok-3 following BCR activation [34]. Thus, phosphorylated Dok-3 provides a ‘membrane zip code’ for Grb2 and the majority of cytosolic Grb2 is recruited to the plasma membrane by this interaction. In DT40 cells that represent an immature B cell stage, Grb2 recruitment is strictly Dok-3-dependent [34]. Dok-3 appears to direct Grb2 into a distinct membrane compartment, where Grb2 forms Dok-3/Grb2 complexes and these complexes inhibits Btk, resulting in decreased PLC-γ2 activity. Recent studies showed mice deficient in Dok-3 exhibit enhanced BCR-induced Ca^2+^_i_ response [32, 34, 36, 37], suggesting that Dok-3/Grb2 complexes also inhibit BCR signaling in mammalian B cells. Honma, *et al*. also showed that overexpression of Dok-3 could inhibit the recruitment of the Grb2/SOS complex [44].

Together with either SOS or Dok-3, CD22/Grb2/PMCA complexes could be generated to enhance PMCA's Ca^2+^_i_-pumping activity. These associations and activity necessary for increasing PMCA function might be very rapid and transitory. Further studies will be required to address how PMCA get activated after the complexes are formed.

Our results integrate the whole image of the negative regulation of BCR-activation-induced Ca^2+^_i_ signal. Grb2 not only attenuates IP_3_ production and subsequent IP_3_-induced Ca^2+^_i_ release (IICR) by forming Dok-3-Grb2 complexes to inhibit PLC-γ2 activity [34], but also potentiates Ca^2+^_i_ extrusion by the CD22/PMCA pathway. Thus Grb2 plays a central role in negative regulation of BCR-activation-induced Ca^2+^_i_ signal. Grb2 has been identified to inhibit Ca^2+^ signaling in both mature and immature splenic B cells isolated from the B-cell-specific Grb2-deficient mice [36, 37], in addition to avian DT40 cells. It is well known that IP_3_ production determines the height of the initial Ca^2+^_i_ spike and less IP_3_ production causes less IICR, which manifests as a lower initial Ca^2+^_i_ spike and less subsequent SOCE [34, 45]. In the absences of Ca^2+^_o_ and SOCE, the sustained phase of Ca^2+^_i_ signaling is mainly determined by PMCA and Na^+^-Ca^2+^ exchanger (NCX) and thus experiment revealing the precise effect of PMCA on the sustained phase are needed to be performed under the blockade of NCX activity [46]. Grb2-dependent inhibition of IP_3_ production and potentiation of PMCA activity could result in an attenuated Ca^2+^_i_ signal, which manifests as a lower initial spike and shortened sustained phase. In this study, transfection of CD22 into Grb2^−/−^ DT40 cells, or rescue of Grb2 in Grb2^−/−^ DT40 cells only attenuated the initial Ca^2+^_i_ spike with some effects on the sustained phase (Figure 4A), similarly to the previous report in mouse Grb2^−/−^ cells [37].

Does the constitutively-existed Grb2/PMCA complex bind only to CD22 to execute their Ca^2+^_i_-extruding function in B cells? Stork *et al*. have compared the Ca^2+^_i_ signal in Grb2^−/−^ DT40 cells with or without rescue of Grb2 [32, 34]. Although both cells demonstrated similar initial Ca^2+^_i_ spikes, Grb2-rescued cells manifested a much shorter sustained phase, indicating that Grb2/PMCA, even in the absence of CD22, is still involved in negative regulation of Ca^2+^_i_ signaling. It is possible that other inhibitory receptors like FcγRIIb, Siglec-G, IgG/IgE could substitute for CD22 to bind and activate Grb2/PMCA function [1, 2, 11, 24, 35].

Our data suggest that Grb2/PMCA is a “tunable” regulator of B cell activation and is essential for B cell development and function. Furthermore, given the ubiquitous expression of Grb2 [31, 32, 34, 36, 37] and the widespread existence of PMCA [47] in both avian and mammalian cells, it is conceivable that the interaction of the Grb2/PMCA complex with other tissue-specific negative regulators may provide a general mechanism for regulating intracellular Ca^2+^_i_ signaling.

## MATERIALS AND METHODS

### Cell culture

The chicken B cell line DT40 (CRL-2111) and the Burkitt's lymphoma cell line Daudi were purchased from ATCC. DT40 cells were cultured in RPMI 1640 medium (Life Technologies) supplemented with 10% heat-inactivated fetal bovine serum, 1% heat-inactivated chicken serum, 2mM L-glutamine, 1 mM sodium pyruvate and nonessential amino acids, 1 mM nonessential amino acids (GIBCO Life Technologies), 50 microM 2-mercaptoethanol, 100 U/ml penicillin, and 100 μg/ml streptomycin. The cells were maintained below a density of 3×10^6^ cells/ml. Daudi cells were maintained by serial passages in RPMI 1640 medium containing 10% heat-inactivated fetal bovine serum and 100 mM L-glutamine. HEK-293T cells were cultured and passaged in Dulbecco's modified Eagle's medium (DMEM; Gibco-Life) supplemented with 4500mg/L high glucose and 10% fetal bovine serum.

### Isolation of normal human peripheral B cells

Healthy blood donors (4 men, 3 women) ranged in age from 42 to 57 years. This study was approved by the Institutional Review Board. Written consent was obtained in each case. Peripheral-blood mononuclear cells were isolated from EGTA-treated venous blood samples by density gradient centrifugation using Ficoll-Paque (Amersham-Pharmacia Biotech, Piscataway, NJ). B cells were isolated from mononuclear cells by CD19 or CD20 selection using immunomagnetic MACS micro-beads (Miltenyi Biotech, Auburn, CA). After isolation, the cells were cultured for a few hours and stimulated with goat anti-human F(ab’)2 IgM (Thermo).

### Molecular cloning and mutagenesis

To construct chicken expression vector (*p*Apuro) harboring the chicken β-actin promoter and puromycin-resistant gene, we used *p*Babe-puro vector (Addgene) as a backbone [48]. The vector contains three *PstI* sites that were used to digest the vector into three fragments. An 872bp fragment between the first and the second *PstI* site containing the Moloney murine leukemia virus long terminal repeat (LTR) was deleted from the vector and a 176bp fragment between the second and the third *PstI* site containing the truncated *gag* was recovered from agarose gel, purified, and re-connected back to the vector. The correct ligation direction was verified by sequencing. A 1283bp fragment containing a chicken β-actin promoter was amplified by PCR using a high-fidelity polymerase KOD-FX-neo (Toyobo, Japan) from chicken genomic DNA [49, 50]. The primers used were β-actin-F: 5′-GGATCCGTGAGCCCCACGTTCTGCTTC-3′ and β-actin-R: 5′-GGATCCGGCTGGCTGCGGAGGAACAGA-3′. Then the PCR fragment was inserted into a unique *BamHI* site of the above modified *p*Babe-puro, resulting *p*Apuro vector [51].

One microgram of total RNA, isolated from either mouse splenic B cells, or DT40 cells, or normal human B cells using TRIzol (Invitrogen), was reversely transcribed into cDNA using Biovisualab RT-kit (Shanghai, China). The full-length mouse CD22 cDNA was amplified by PCR using KOD-FX-neo with primers: mCD22-F primer (5′ -CCCAGTGTGGTGGTA CGTAGGAATTCATGCGCGTCCATTACCTGTGGC - 3′) and mCD22-R primer(5′-ACTGACACACATTCCACAGGGTCGACTCAGTGCTTGAGGGTCACATAGT - 3′). The PCR product was cloned into the *p*Apuro between *EcoRI*-*Sal*I sites using GBI-clonart seamless ligation kit (Genbank Bioscience, Suzhou, China). Obtained *p*Apuro-CD22 constructs were sequenced and matched with Genbank sequences (CD22: NM_001043317.2).

The CD22 mutants with cytoplasmic tyrosines changing to phenylalanine were made in multiple steps. *ApaApa*we amplified a 320bp fragment from a CD22 cytoplasmic far C-terminus by overlap extension PCR using various mutagenic primers. The forward primers contain the 5′-*Apa I* site and the reverse primers contain the 5′-*SalI* site. The PCR products with mutations of tyrosine to phenylalanine, or asparagine to aspartic acid, or both, were cloned back into *p*Apuro-CD22 construct with *ApaI-SalI* double cuts. The resultant mutants were selected by sequencing (Figure 1B).

The full-length chicken Grb2 cDNA were obtained by PCR using cGrb2-F primer(5′-CCCAGTGTGGTGGTACGTAGGAATTCATGGAAGCCATCGCCAAATAC - 3′) and cGrb2-R primer (5′ -ACTGACA CACAGGGTCGACCTAGATGTTCCGGTTCACTG - 3′). The PCR product was cloned into the *p*Apuro vector between *EcoRI*-*SalI* sites. The full-length human Grb2 cDNA was obtained by PCR using the hGrb2-F primer (5′- TGGTTCCGCGTGGAT CCCCGGAATTCATGGAAGCCATCGCCAAATATG - 3′) and hGrb2-R primer (5′ -GATCGTCAGTCAGTCAC GATGCGGCCGCTTAGACGTTCCGGTTCACGG - 3′) and was cloned into the *p*GEX-4T-1 between *EcoRI*-*NotI* sites and the *p*ET-28A(+) between *BamHI*-*XhoI* sites. The full-length human PMCA4b (3618bp) cDNA were obtained by PCR using the hPMCA4b-F primer (5′ -AGCTGGCTAGTTAAG CTTGGTACCAAAATGACGAACCCATCAGACCG - 3′) and hPMCA4b-R primer (5′ -TTTTTGTTCGAAGG GCCCTCTAGAAACTGATGTCTCTAGGCTCTG - 3′) and was cloned into the *p*cDNA3.1-myc/his(+) between *KpnI-XbaI* sites. The N-terminus (NT; AA 1-92), the first (Loop-1; AA 172-356) and second (Loop-2; AA 428-840) cytoplasmic loops, and the C-terminus (CT; AA 1048-1241) of the human PMCA4b, were amplified by PCR from the full length cDNA and ligated into *p*GEX-4T-1 between *EcoRI*-*NotI* sites. All constructs were fully sequenced in both directions and matched with Genbank sequences (chicken Grb2: EF062570.1; human Grb2: NM_002086.4; human PMCA4b: NM_001684.4).

### Plasmid and siRNA transfection

DT40 cells were transfected with mouse CD22-WT, CD22 mutants, or chicken Grb2 a Gene Pulser II electroporation system and the Capacitance Extender Plus module (Bio-Rad) according to the manufacturer's instructions. Stable transfectants were selected in 0.5 μg/ml puromycin and puromycin-resistant clones with similar expression of CD22 and surface IgM, as measured by flow cytometry, were used for study. siRNA for human CD22 were synthesized by RiboBio (Guangzhou, China) (CD22-1: 5′-GGAAGUUCCUCUCCAAUGAdTdT-3′, CD22-2: 5′-CAUGCCGAUUCGAGAAGGAdTdT-3′). Daudi cells cells were transfected with siRNA for 72~96 hours. HEK-293T cells were transfected with plasmid for 48 hours [52, 53].

### GST- and 6His-fusion protein expression, purification and pull-down assay

All GST- and 6His-fusion proteins were expressed in BL-21 cells by induction of isopropyl-D-thiogalactopyranoside and purified through glutathione-sepharose beads (Sigma G4510) or Ni-beads (Qiagen, Cat-70666), as described previously [54]. 6His-fusion proteins were eluted from Ni-beads with 250mM imidazole and followed by a dialysis. HEK293 cells over-expressed with PMCA4b were lysed in TLB buffer (20 mM Tris-HCl, 137 mM NaCl, 2 mM EDTA, 10% glycerol, 1% Triton X-100, 25 mM β-glycerol phosphate, 0.01μg/ml each of aprotinin, leupeptin and pepstatin A, 2mM phenylmethylsulfonyl fluoride, pH 7.4) and microfuged. The supernatant, or purified 6His-fusion proteins, was mixed with GST or GST-fusion proteins in TLB buffer, rocked for 2 hour at 4°C. The beads were then washed three times with TLB buffer, and boiled in SDS sample buffer. The bound proteins were resolved by SDS-PAGE and immunoblotted with the indicated antibodies.

### Antibodies

The Anti-mouse CD22 antibody were purchased from BD (BD Biosciences Pharmingen) [39]. The polyclonal antibodies against Grb2 were purchased from Santa Cruz (C-23) and monoclonal antibodies against Grb2 were from BD. The anti-PMCA mouse monoclonal antibodies 5F10 and JA3 were obtained from Abcam (ab2825) and Santa Cruz (SC-20027) respectively.

### Measurement of [Ca^2+^]_i_

Cells (2 × 10^6^) were incubated with 2 μM Fura-2AM (Molecular Probes) for 30 min at 37°C in Fura-2 buffer [135mM NaCl, 1mM CaCl_2_, 1mM MgCl_2_, 5mM KCl, 10mM glucose, 10mM HEPES, and 0.1%BSA (Sigma), pH 7.4]. Fura-2 buffer was also used as extracellular medium for measuring [Ca^2+^]_i_. The Ca^2+^-free buffer consisted of Fura-2 buffer with CaCl_2_ replaced by 1mM EGTA. Fluorescence from cells in suspension was monitored with a QuantaMaster Series fluorometer (PTI, USA) at excitation wavelengths of 340 nm and 380 nm and an emission wavelength of 510 nm. Anti-chicken IgM M4 or anti-human IgM (1~10 μg/ml) was used to activate BCR on B cells [14]. Because the fura-2 method has several intrinsic problems in the estimation of absolute [Ca^2+^]_i_ [55], the amplitude of Ca^2+^_i_ elevation in response to BCR activation was calculated using the percentage of increase of F340/F380 with reference to F340/F380 at the resting state. To quantify the Ca^2+^_i_ responses, we measured five parameters [56]: Ca^2+^_i_ peak level, Δpeak ( = CD22-mutant's Ca^2+^_i_ peak - CD22-WT's Ca^2+^_i_ peak), the area under the Ca^2+^_i_ curve (AUC), ΔAUC (CD22-mutant's AUC - CD22-WT's AUC), and %ΔAUC ([CD22-mutant's AUC - CD22-WT's AUC] / [Empty vector's AUC - CD22-WT's AUC] x100%) by computer-assisted planimetry. Of the five, Δpeak and %ΔAUC are two key parameters.

### Measurement of extracellular Ca^2+^ ([Ca^2+^]_o_)

[Ca^2+^]_o_ was measured according to the previous reported method [39]. In brief, [Ca^2+^]_o_ was measured with 0.024 μM Fura-2 (cell-impermeable form; Molecular Probes). Cells (3×10^6^) were suspended in 2 ml efflux buffer and [Ca^2+^]_o_ brought to ~160nM with EGTA (pH 8.0). Efflux buffer contains 135mM NaCl, 1mM MgCl_2_, 5mM KCl, 10mM glucose, 10mM HEPES, 3mM EGTA, 0.1% BSA, pH 7.4. Similarly, the amplitude of [Ca^2+^]_o_ elevation in response to BCR activation was calculated using the percentage of increase of F340/F380 with reference to F340/F380 at the resting state. The percentage at 300 seconds after BCR stimulation was set as a point for comparison between groups.

### Immunoprecipitation and immunoblotting

Cells were solubilized in 1% NP-40 lysis buffer (137mM NaCl, 10% glycerol, 1% NP-40, 20mM Tris-HCl, pH 7.5) with a proteinase inhibitor mixture (1 μg/ml aprotinin, 1 μg/ml leupeptin, 1 μg/ml pepstatin A, 1 mM phenylmethylsulfonyl fluoride; all from Sigma). Lysates were incubated sequentially with appropriate antibodies and protein A/G-agarose (Roche). The immunoprecipitants were washed four times with lysis buffer, boiled in SDS-sample buffer, and resolved by electrophoresis in a 7~12% SDS-PAGE, followed by blotting onto polyvinylidene difluoride membranes (Millipore). After overnight incubation at 4°C with primary antibodies (1:1000, otherwise noted), blots were washed 4 times in PBS containing 0.05% Tween-20 and then incubated with HRP-conjugated secondary antibodies (1:1000 dilution) for 1 hour at room temperature before extensive washes. The blots were visualized using an ECL detection kit (Thermo-Pierce) on the Chemi Doc^TM^ XRS^+^ (Bio-Rad).

### Knockout of chicken Grb2 gene by CRISPR/Cas9-gRNA

The Grb2-specific guide RNA (gRNA) sequence preceding the PAM motif for CRISPR/Cas9 interference were designed from the region of the exon-1 of the chicken Grb2 gene (Figure 1S) and were tested to avoid obvious potential off-target effects by bioinformatics analysis (http://www.e-crisp.org) [53, 57]. The pVK-001-08 plasmid with avian promoter cU6 was purchased from Weishanglide (Beijing, China; Figure 1S). The gRNA sequence was obtained by annealing two primers (Target-F: AAACACCG-GACGAGCTGAGCTTCAAA; Target-R: CTCTAAAAC-TTTGAAGCTCAGCTCGTC) in the buffer: 40 mMTris-HCl, 20 mM MgCl2, 50 mM NaCl, pH 8.0. The annealed oligonucleotides were inserted into the pre-cut pVK-001-08 and was transformated into E. coli (XL-10-gold). The correct pVK-Grb2 construct was selected by sequencing.

DT40 cells at a density of 2×10^5^ cells/ml in a 6-well plate were transfected with 2.5 μg of plasmid pVK-Grb2 by electroporation. Twelve hours post-transfection, media was refreshed. At 72 hours, the cells were re-seeded into a 10 cm dish with 1 μg/mL puromycin. The media with antibiotics was changed every 2 days. Upon complete cell death in the controls (untransfected cells with single puromycin selection) after a week of selection, cells were plated in two 96-well plates at a density of 0.3 cells per well to isolate single cell derived clones. Wells were monitored for single clone expansion. Upon observation, the expanded clone was divided into two parts, half for freezing and half for genomic DNA extraction.

Genomic DNA from transfected and control DT40 cells was extracted using a genome isolation kit (Promega). The genomic fragment was obtained by PCR using KOD-FX-neo and primers (Grb2-Exon1-F: 5′-ACTTCCCCTAATGGCACGGC-3′ and Grb2-Exon1-R: 5′-CCAACATTTGACAGCCTGATG-3′) under the following conditions: 94°C for 2min; 35 cycles (94°C for 40s, 57°C for 30s, 68°C for 45s). The fragments from both transfected and control cells were purified, mixed in equal amount, and subjected to re-annealing reaction. Re-annealed PCR products were incubated for 30 min at 37 °C in the presence of 10 U of mismatch-sensitive T7 endonuclease I (New England Biolabs) and analyzed by agarose gel electrophoresis. Monoallelic deletion clones were defined as having both the non-deletion band and deletion band. Biallelic deletion clones were defined as having the deletion band and absence of the non-deletion band. Having the non-deletion band and absence of the deletion band were defined as non-deletion clones (Figure 1S). By using this assay, two clones with biallelic deletion were selected. They were further expanded and the genomic DNA was extracted. The genomic fragments obtained by PCR once again were purified and directly sequenced with primer 5′-TGTGGAAGGGAATTCAGCAC-3′. They were also checked for the effect of Grb2 knockout by *Western* blotting.

### Statistical analysis

All data are given as the mean±S.E. Data were analyzed by using a paired or unpaired Student's *t* test. *P* < 0.05 was taken as a significant difference between data sets.

## SUPPLEMENTARY MATERIAL FIGURES



## Abbreviations

AA: amino acid
BCR: B cell antigen receptor
bp: base pair
Ca^2+^_i_: intracellular Ca^2+^
[Ca^2+^]_i_: intracellular Ca^2+^ concentration
Ca^2+^_o_: extracellular Ca^2+^
Cas9: CRISPR associated 9
Co-IP: co-immunoprecipitation
CRISPR: clustered regularly interspaced short palindromic repeats
FcγRII: low-affinity Fc receptor for IgG
Grb2: growth factor receptor-bound protein 2
IB: immunoblot
IP_3_: inositol-1,4,5-trisphosphate
IP: immunoprecipitation
ITIM: immunoreceptor tyrosine-based inhibition motif
Lyn: lck/yes-related protein tyrosine kinase
PLC-γ2: phospholipase C-γ2
PMCA: plasma membrane calcium-ATPase
SH2: Src homology 2 domain
SHIP: Src homology 2 (SH2)-containing inositol 5′-phosphatase
SHP-1: Src homology 2 domain-containing tyrosine phosphatase-1
SOCE: Store-operated Ca^2+^ entry
SOS: Son of sevenless
WT: wild type
Y: tyrosine
pY: phosphotyrosine.
