# Supplementary material for: Besides an ITIM/SHP-1-dependent pathway, CD22 collaborates with Grb2 and plasma membrane calcium-ATPase in an ITIM/SHP-1-independent pathway of attenuation of Ca2+i signal in B cells
Source: Oncotarget. 2016 Jun 2;7(35):56129–46. doi: 10.18632/oncotarget.9794 (PMC5302901; doi:10.18632/oncotarget.9794)
Supplement: Supplementary file 1 [file oncotarget-07-56129-s001.pdf]

Besides an ITIM/SHP-1-dependent pathway, CD22 collaborates with Grb2 and plasma membrane calcium-ATPase in an ITIM/SHP-1-independent pathway of attenuation of $Ca^{2+}{}_i$ signal in B cells

Supplementary Material

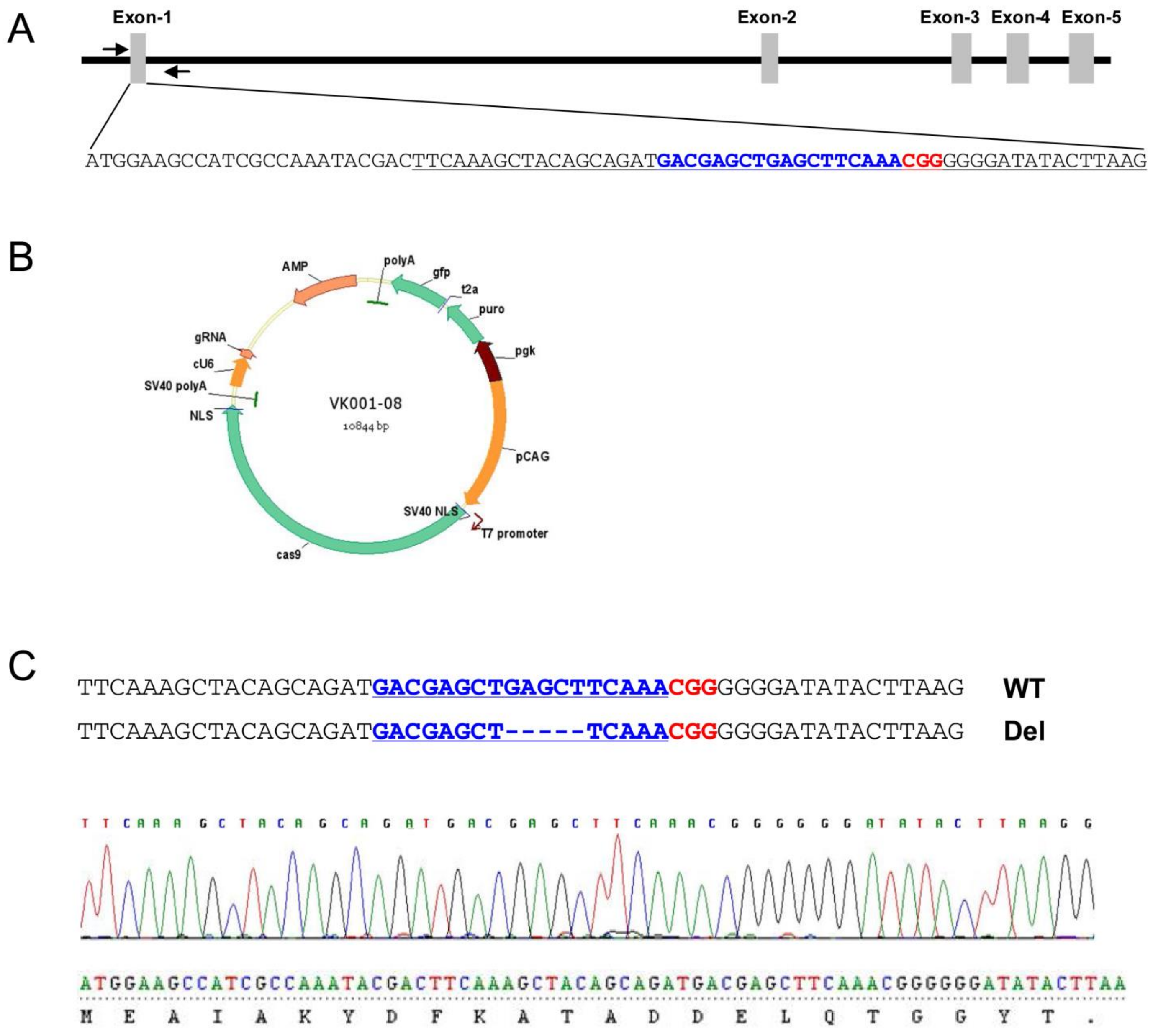

Supplementary Figure 1. Cas9/gRNA-induced mutations at the Grb2 locus. Figure *A* shows chicken Grb2 gene exons in boxes and *arrows* represent the primers used to amplify the target region. Target region is highlighted in bold and blue and PAM sequences are illustrated in red among the exon-1. Figure *B* illustrates the map of VK001-08 vector which is equipped with avian cU6 promoter. Figure *C* demonstrates the sequences of deletion shown in hyphens in clone *Del*. The sequence at bottom shows the deletion results in a frame shift.
